# Structural Study of Heterogeneous Biological Samples by Cryoelectron Microscopy and Image Processing

**DOI:** 10.1155/2017/1032432

**Published:** 2017-01-15

**Authors:** H. E. White, A. Ignatiou, D. K. Clare, E. V. Orlova

**Affiliations:** Institute of Structural and Molecular Biology, University College London and Birkbeck, Malet Street, London WC1E 7HX, UK

## Abstract

In living organisms, biological macromolecules are intrinsically flexible and naturally exist in multiple conformations. Modern electron microscopy, especially at liquid nitrogen temperatures (cryo-EM), is able to visualise biocomplexes in nearly native conditions and in multiple conformational states. The advances made during the last decade in electronic technology and software development have led to the revelation of structural variations in complexes and also improved the resolution of EM structures. Nowadays, structural studies based on single particle analysis (SPA) suggests several approaches for the separation of different conformational states and therefore disclosure of the mechanisms for functioning of complexes. The task of resolving different states requires the examination of large datasets, sophisticated programs, and significant computing power. Some methods are based on analysis of two-dimensional images, while others are based on three-dimensional studies. In this review, we describe the basic principles implemented in the various techniques that are currently used in the analysis of structural conformations and provide some examples of successful applications of these methods in structural studies of biologically significant complexes.

## 1. Introduction

Biological molecular assemblies are dynamic machines that can adopt different conformations (local positions) of their domains or subunits in order to perform their functions in the cell. Even when these molecules are purified in vitro, they can be flexible and adopt various possible spatial arrangements of domains in a biocomplex. The multitude of different states is typically identified as sample heterogeneity. Moreover heterogeneity can also arise in vitro due to differences in buffer, temperature, variable ligand binding, and interactions between molecules or different types of oligomers. For example, a virus sample may contain virions in different stages of maturation [1]; ribosome samples may have subunits in different orientations since they have to move to synthesise polypeptide chains according to the messenger RNA, and a nascent polypeptide chain may have a variety of “prefolding” states within the exit tunnel of ribosomes [2–4]; chaperones are another example of active machines engaged in the dynamic process of refolding substrate molecules and can adopt different conformations during their reaction cycle [5, 6].

X-ray crystallography is a classical technique for determining atomic structures of proteins and protein complexes and relies on the high homogeneity and stability of the sample being crystallised. Often, to facilitate crystallisation proteins may need to be modified in such a way that their flexible regions are removed or substrates are added to stabilize the molecules [7–9]. Consequently, what is seen in a crystal structure may not always be a truthful representation of what is happening in vivo and does not necessarily reflect the biologically active native form. Structural studies using cryoelectron microscopy (cryo-EM) offer methods for examination of molecules/protein complexes in near-native conditions as no crystal needs to be formed [10–13]. In cryo-EM sample molecules are trapped in frozen vitrified solution in nearly native environment at liquid nitrogen temperatures. This technique has improved rapidly over the last few years and is now able to achieve 2.5–4 Å resolution, allowing amino acids of the polypeptide chains to be seen [14–17].

**Figure 1:**
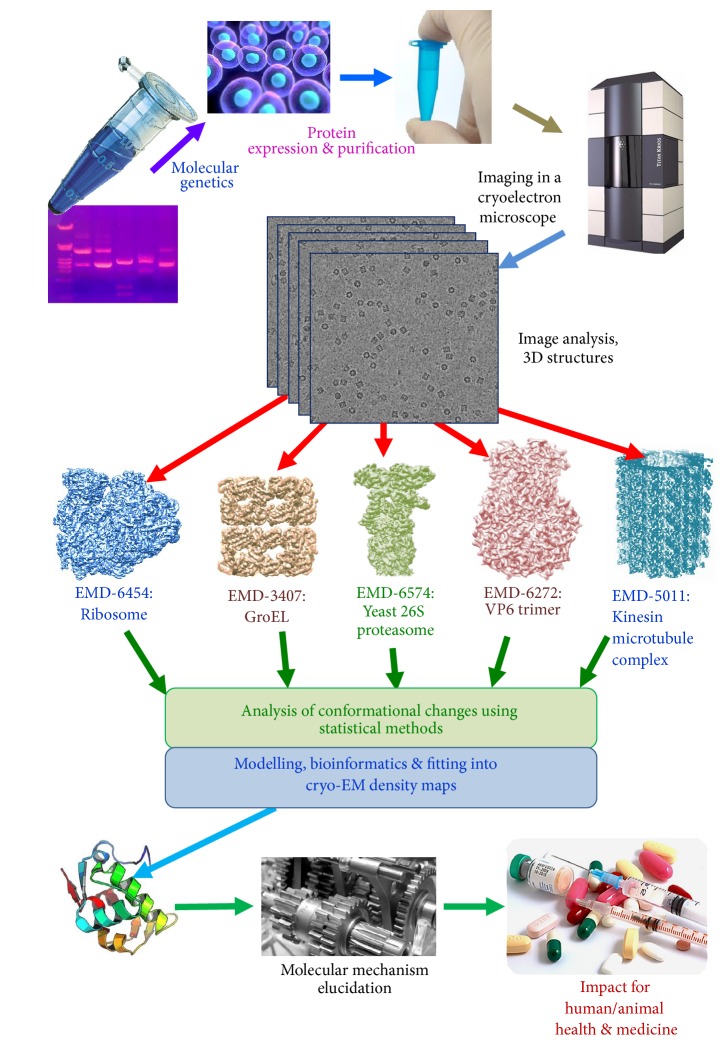
Overall diagram of the work flow of structural analysis by cryo-EM.

Structural studies using EM are based on imaging of the protein complex followed by a sophisticated computational process (Figure 1). It starts with the automated data collection on the microscope, correction for the distortions present in the recorded images often induced by the microscope and recording systems, separation of characteristic views of the imaged proteins, and eventually reconstruction of a three-dimensional distribution of electron densities of the protein complex [20]. The electron density maps are then interpreted using methods that dock and refine atomic or homology models or by building de novo atomic models [21–23]. However, if there is significant heterogeneity present in the sample, the electron density may not be well defined in certain areas of the map or may affect the entire density distribution. This will not allow an unambiguous interpretation of the protein complex map. In some samples heterogeneity is clearly visible in EM images, particularly if there is a significant size difference, for example, if a large substrate is not stably bound to the outer surface of a complex. However, if the changes are small or they take place inside the complex, they will be difficult to identify and may cause the structure not to refine. Such heterogeneity limits the level of detail revealed in structures, as the information from the different conformations will be averaged out in the final reconstruction. This is why various approaches are used to trap biomolecular complexes in different states. An example of this is the ribosome where antibiotics such as kirromycin, sordarin, and others were used to stall the process of protein translation [24–28]. Mutagenesis of the protein has also has been used to produce more stable complexes by removing the flexible regions, which is a standard approach in X-ray crystallography to form good crystals. However, it is not always possible to biochemically trap the most biologically interesting conformations. Several computational techniques in electron microscopy were developed to overcome the problem of sample heterogeneity. All of them are based on statistical approaches that analyse large datasets of particle images. A combination of biochemical methods that will allow complexes to be trapped in a limited range of conformations, together with statistical methods of image analysis, could allow us to link conformations observed in the structures to the movements and specific features in the function of the biological complex [29–31].

Another problem intrinsically linked to the EM imaging of biological molecules is that images in EM are formed by electrons and are registered nowadays with the help of digital cameras. Since biological samples should be preserved in the vacuum system of the microscope they have to be fixed with negative stain or frozen in a thin layer of vitrified ice [20]. These conditions and systems of recording lead to a high level of noise in the images. Another reason for image degradation is beam induced movement. The use of direct electron detectors has helped increase the quantity and improve the quality of the images that we can collect and use. EM images are now recorded as multiple frames by the new direct detectors and these frames can be aligned eliminating the effect of initial strong movement of samples and effects of drift. The averaged image after alignment of subframes removes the noise associated with beam induced movement and low dose [54]. Movie mode processing in combination with the improved performance of the new detectors over all spatial frequencies in the image have now become a standard procedure to obtain higher resolution structures [14, 55–58].

Improvements in technology and image quality have dramatically expanded the capacity of structural analysis by cryo-EM thus not only enabling visualisation of different conformations but also revealing ligands on an atomic level [59, 60]. However, these results did not come at the same time. Development of methods to analyse heterogeneity has taken several decades. The first two methods developed were multivariate statistical analysis (MSA) [61] and principle component analysis (PCA), [62, 63], both of which were initially mostly used to distinguish different views of the same complexes. Later the maximum likelihood (ML) method has been implemented in electron microscopy [45, 46, 64, 65]. Originally these techniques were used to analyse two-dimensional (2D) images but later they have been used in the analysis of three-dimensional (3D) EM maps. During the last decade the bootstrap method and covariance analysis were also used to analyse sample heterogeneity [66, 67]. A number of other papers on statistical methods have been published recently [28, 51, 68–71]. New developments are based on increasingly improved speed of calculations and new multiprocessor technology. Here we aim to provide a review of different statistical methods used in the analysis of both 2D projection images and 3D maps. However, it should be noted that new approaches are still evolving, new algorithms being proposed, and currently the reader will be provided with a snapshot of the latest developments.

## 2. Theoretical Background

### 2.1. Basic Concepts Used in Statistical Analysis

Unfortunately images of biocomplexes recorded in EM are obscured by noise for different reasons. Noise in images is caused by irregularities in the distribution of the negative stain grain used during sample preparation, buffer distribution, variations in ice thickness in cryopreparations, and low dose conditions where one reduces the electron dose to avoid radiation damage of the sample but this leads to a small number of electrons forming the image. Also beam induced movement/drift of biomolecules is a reason that images became blurry [72, 73]. If the sample has been applied on a carbon film it adds significantly to the level of noise and reduces the intensity of information related to the biological molecule. This has more of a negative effect on the imaging of small complexes with a molecular weight of less than ~350 KDa. Another reason for variation in images, which is the most interesting part in these studies, is the existence of the biocomplexes in different phases of their functional action. Now in the era of direct electron detectors, which have significantly improved the recording quality of images compared to the old CCD detectors [55], the problem of differentiating a real signal from noise is still important due to specific features of their sensors [74, 75]. In order to obtain a characteristic view of the molecule, one has to find similar images and then average them to increase the signal-to-noise ratio. With thousands of different particle images it is a challenge to deduce the best criteria according to which particles should be grouped together. A researcher has to firstly remove the effects of noise and distortions in the images and then identify differences in the images due to conformational variations.

### 2.2. How the Signal Is Related to the Images

The sources of noise mentioned above are not dependent on the features of the biocomplexes in the study and therefore the noise $N\left(\vec{r}\right)$ (noninformative signal) is considered as random, uncorrelated to the signal (meaningful information), and additive. So an image $I\left(\vec{r}\right)$ represents a projection $S\left(\vec{r}\right)$ of a bioparticle, where $\vec{r}$ is a vector indicating a point in the image and $N\left(\vec{r}\right)$ is additive noise at the same point:

(1) $$\begin{array}{c} I\left(\;\vec{r}\;\right)=S\left(\;\vec{r}\;\right)+N\left(\;\vec{r}\;\right). \end{array}$$

The necessity to collect data at very low electron doses in order to avoid radiation damage and factors related to the low contrast of complexes in images and high sensitivity of digital detectors mean that the signal-to-noise ratio (SNR) is very low. There are several definitions of SNR that are not completely equivalent [76]. In imaging the SNR is defined as the ratio of the* mean value* of the signal and the* standard deviation σ*_noise_ of the noise $N\left(\vec{r}\right)$.

(2) $$\begin{array}{c} SNR=\frac{S_{avr}}{\sigma_{noise}}. \end{array}$$

We assume that noise has an average value equal to zero. To fulfil our task for determination of biocomplex structures from images of single particles we need to improve the signal and reduce the noise in order to make the SNR bigger. Averaging of similar images improves the SNR. If we have the same complex imaged *L* times (we assume that the particle is in the same orientation) the signal component is the same at each measurement. It means that images $S_{i}\left(\vec{r}\right)$ are the same and equal to $S\left(\vec{r}\right)$:

(3) $$\begin{array}{c} S_{avr}=\frac{1}{L}\overset{L}{\underset{i=1}{\sum }}S_{i}\left(\;\vec{r}\;\right)=S\left(\;\vec{r}\;\right),\;where\;\;i=1,2,\ldots ,L. \end{array}$$

During registration of images we make another assumption that noise components $N_{i}\left(\vec{r}\right)$ are not correlated to each other or to the signal and have the same standard deviation *σ*_noise_ in all registered images. The result of averaging of *L* images can be defined as follows:

(4) Iavr1L∑i=1LIir→=1L∑i=1LSir→+1L∑i=1LNir→=Sr→+Navr.

Since noise is random, therefore *σ*_noise_avr_ after summation of *L* images is defined as

(5) $$\begin{array}{c} \sigma_{noise\_avr}=\sqrt{\frac{1}{L}\overset{L}{\underset{i=1}{\sum }}{\left(N_{i}\left(\vec{r}\right)\right)}^{2}}=\frac{1}{\sqrt{L}}\sigma_{noise}. \end{array}$$

Then the SNR will be

(6) $$\begin{array}{c} {SNR}_{avr}=\sqrt{L}*SNR. \end{array}$$

The result of summation of *L* images leads to the improvement of the SNR $\sqrt{L}$ times, where *L* is the number of images. However, before averaging, images have to be aligned and evaluated for similarity, since nonaligned and different images will result in the loss of information.

### 2.3. Concept of the Correlation Function

A low signal-to-noise ratio in EM images of vitreous specimens makes it difficult to see differences in the size and orientation of single images of the particles. However, determination of the particle orientations in images is crucial for 3D analysis. To answer the question “does a set of images represent a biocomplex in the same or different orientations?” one needs to assess their likeness. A general method to assess the similarity of two objects $F\left(\vec{r}\right)$ and $G\left(\vec{r}\right)$ (images) is to use a cross-correlation coefficient (CCF), which is defined as a measure of similarity of two functions. The functions can be multidimensional, where the variable $\vec{r}$ is a multidimensional vector and ${\vec{r}}^{\prime }$ is a shift of the function $G\left(\vec{r}\right)$ with respect to the function $F\left(\vec{r}\right)$. To assess the level of similarity, one has to multiply the two functions point by point, and the results of each multiplication are then summed; this operation is performed for different shifts. The location of the maximum of this new CCF function which depends on the shifts will give information on how one image $G\left(\vec{r}\right)$ is displaced with respect to the image $F\left(\vec{r}\right)$ and the height of the output correlation peak indicates the degree of their similarity. The CCF should be normalized using the product obtained from the multiplication of each function by itself.

(7) $$\begin{array}{c} CCF\left(\;{\vec{r}}^{\prime }\;\right)=\frac{\int F\left(\vec{r}\right)G\left({\vec{r}}^{\prime }+\vec{r}\right)d\vec{r}}{\sqrt{\int F\left(\vec{r}\right)F\left(\vec{r}\right)d\vec{r}\int G\left(\vec{r}\right)G\left(\vec{r}\right)d\vec{r}}}. \end{array}$$

The height of the CCF maximum serves as a measure of the image similarity and is named as the cross-correlation coefficient (CCC). If images are identical then the CCC is equal to 1. The value of ${\vec{r}}^{\prime }$ where the CCF has the maximum indicates the coordinates of the best correspondence between the two images. Images can then be sorted using the CCF between all possible pairs to assess similarities and differences, a task that is not difficult until one has tens of thousands of images and at that stage it becomes computationally expensive.

## 3. Multivariate Statistical Analysis

### 3.1. Principles of MSA

Work in the EM field using multivariate statistical analysis (MSA) was initiated by van Heel and Frank in 1981/1982, who combined their efforts to solve the problem of recognising/distinguishing characteristic (reliable) views in negatively stained samples with MSA. It was used to find variations due to differences in structure rather than those due to different orientations [77–79].

The main advantage of multivariate statistical analysis (MSA) is its ability to examine relationships among multiple variables at the same time. Different versions of this analysis have been implemented, but all are based on reducing the number of variables in such a way that only the most significant ones are used. The question is how to find the essential variables (parameters) and to avoid the influence of unimportant parameters such as noise. One of the most helpful descriptions of MSA has been given by van Heel and coauthors [80].

**Figure 2:**
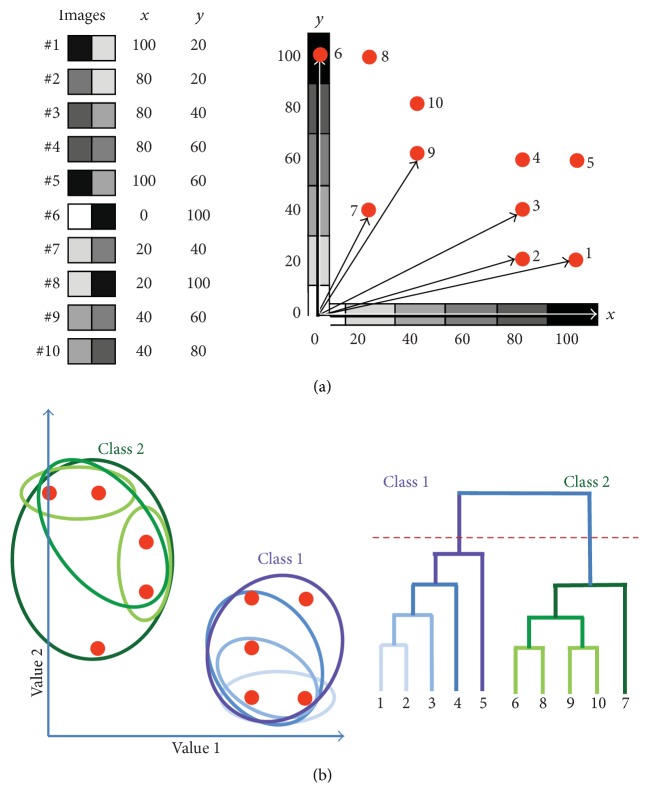
*Multivariate Statistical Analysis.* (a) Left: ten images, each consisting of 2 pixels. Right: each image is represented as a vector in 2-dimensional space according to their grey values. (b)* Hierarchical Classification.* The left panel shows the sequential combination of vectors according to their closeness. The initial classification of images starts by forming small classes which include images that are close to one another in multidimensional space and then the size of the group is progressively increased by merging with dimensional other surrounding smaller groups that are in close proximity to each other (see the text). Images that are too far from each other form new separate classes. In the example shown in panel (b) the process of forming two classes is represented by the blue and green ovals which have varying degrees of colour intensity. The light and dark coloured ovals correspond to the initial and final steps of classification, respectively. The right panel shows a tree of HAC. The starting point is 10 classes which correspond to the number of single images in the dataset. The cut-off point is shown by the dashed red line if 2 classes are required and this corresponds to the two classes shown in the left panel.

An image (or a 3D volume or an object from statistical dataset) can be considered as a point (or more correctly as a vector) in multidimensional space, where its coordinates are defined by the grey values (intensities) in each one of its pixels ((or voxels) Figure 2(a), left). One image would correspond to one vector in such a space. If the images are formed only by two pixels we will get 2-dimensional space, and we will be able to show it as a figure, but a higher dimensionality which is equal to the number of pixels would be difficult to illustrate. If one has 10 such images, then there will be 10 different vectors that have two coordinates (Figure 2(a), right). Therefore the comparison of the 10 images can be considered as the comparison of these ten vectors, the ends of which form a data cloud (see [80]). The images or volumes that are similar to each other will form a cluster (a class) of vectors with their ends in close proximity to each other; these small differences are typically induced by noise (Figure 2(b), left). However, if the distances between the vector ends are large (compared with the length of the vectors) or they make another cluster of points, sufficiently remote from the first one, they could represent a group of images (or volumes) that have different features related to conformational changes or from a different angular projection (Figure 2(a), right). The essence of the MSA approach is in the assessment of variations within the cloud of points and the determination of variations which are significant or not. These variations can be ranked according to the distances found between points representing the dataset. Categorized variations are used as a new system of coordinates for the entire dataset and using only the most significant one of them leading to the reduction of variables taken into consideration during analysis. This allows us to concentrate on the most important variations found in the dataset and to ignore sources of insignificant variability (typically related to noise in images).

**Figure 3:**
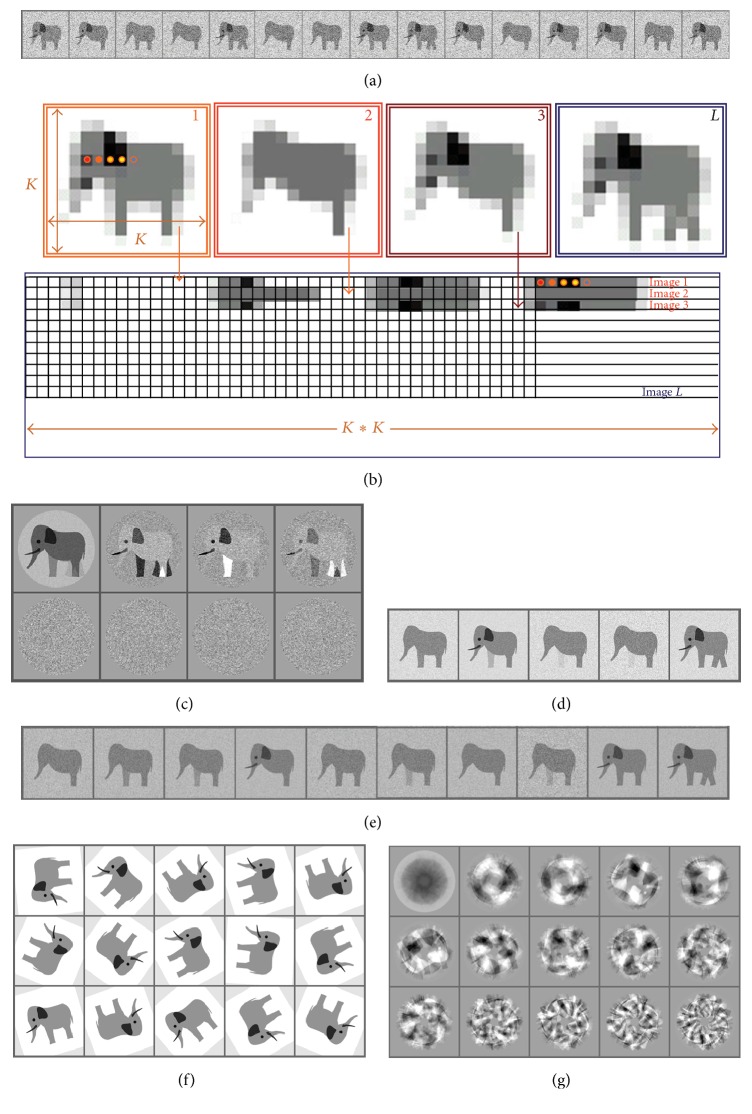
*Eigenimages and Classification.* (a) A set of raw images. (b) Four images (top) shown with a coarse pixilation similar to those in panel (a) with size *K* × *K* pixels. Images form a matrix where a single image is presented as a single row in it (bottom). Each pixel in row 1 of image 1 is laid out in the first row of the matrix. The second row of image 1 follows on after row 1 in the first row of the matrix. This continues until all *K* rows have been laid out in the first row of the matrix. The rows of image 2 are laid out in a similar manner in row 2 of the matrix and the process continues until all *N* images in the dataset have been placed into the matrix. (c) Eight eigenimages obtained from the set of aligned images in (a). (d) Classification of the dataset into 5 classes. (e) Classification of the dataset into 10 classes. (f) Raw unaligned rotated images. (g) Eigenimages from the unaligned dataset.

How can one do such an estimation of variations for large datasets? Mathematically the entire dataset can be represented as a matrix** D** where each line corresponds to one image and its length is defined by the size of the image (or a volume; see Figures 3(a) and 3(b)). The number of lines corresponds to the number of images. However, the number of images *L* is often less than the number of variables in each image *K∗K* which makes the matrix** D **not square. Having so many variables the problem of comparison of images can be solved by determination of eigenvectors of the covariance matrix **C** which is defined as [81]

(8) $$\begin{array}{c} C=D^{T}*D-{\vec{O}}^{T}*\vec{O}, \end{array}$$

where $\vec{O}$ is a vector representing the average of all images in the dataset, **D**^*T*^ is transpose of the matrix **D**, and ${\vec{O}}^{T}$ is a transpose of the vector $\vec{O}$:

(9) $$\begin{array}{c} C{\vec{V}}_{j}=\gamma_{j}{\vec{V}}_{j}. \end{array}$$

If the vectors ${\vec{V}}_{j}$ multiplied on matrix** D **scale the matrix by coefficients *γ*_*j*_ (scalar multipliers) then these vectors are termed as eigenvectors, and scalar multipliers are named as eigenvalues of these characteristic vectors.

The eigenvectors reflect the most characteristic variations in the image population [78, 80, 82]. Details on eigenvector calculations can be found in van Heel et al., 2016 [80]. The eigenvectors (intensity of variations in the dataset) are ranked according to the magnitude of their corresponding eigenvalues in descending order. Each variance will have a weight according to its eigenvalue. Representation of the data in this new system coordinates allows a substantial reduction in the amount of calculations and the ability to perform comparisons according to a selected number of variables that are linked to specific properties of the images (molecules).

MSA allows each point of the data cloud to be represented as a linear combination of eigenvectors ${\vec{V}}_{i}$ with certain coefficients *A*_*i*_. The number of eigenvectors *J* used to represent a statistical element (the point or the image) is substantially smaller than the number of initial variables in the image.

(10) $$\begin{array}{c} I\left(\;\vec{r}\;\right)=A_{1}\vec{V_{1}}+A_{2}\vec{V_{2}}+\cdots +A_{J}\vec{V_{J}}, \end{array}$$

where *J* ≪ *K∗K* and *K* is the image size.

Clustering or classification of data can be done after MSA in several ways. The Hierarchical Ascendant Classification (HAC) is based on distances between the points of the dataset: the distances between points (in our case images) should be assessed and the points with the shortest distance between them form a cluster (or class), and then the vectors (their end points) further away but close to each other form another cluster. Each image (the point) is taken initially as a single class and the classes are merged in pairs until an optimal minimal distance between members of a single class is achieved, which represents the final separation into the classes. The global aim of hierarchical clustering is to minimize the intraclass variance and to maximize the interclass variance (between cluster centres) (Figure 2(b), right). A classification tree contains the details of how the classes were merged. There are a number of algorithms that are used for clustering of images. Since it is difficult to provide a detailed description of all algorithms in this short review, the reader is directed to some references for a more thorough discussion [63, 80, 83–85]. In Figure 2(b), 10 classes (corresponding to a dataset of 10 single images) have been chosen at the bottom of the tree and these have been merged pairwise until a single class is reached at the top of the tree (Figure 2(b)). The user can then decide on the number of classes and thus where the tree will be cut.

Another idea of separation of images into classes is based on the opposite concept, where initially all data points are considered as one class and the distances of each data point from the centre of the cluster are assessed and the class is separated into two where the points are closer to each other (divisive hierarchical clustering). It should be noted in EM that agglomerative algorithms are mostly used. Both procedures are iterative which is continued until there is no movement between the class elements.

In 2D clustering analysis (CL2D) Sorzano and coauthors suggested the use of correntropy as a similarity measure between images instead of the standard least-squares distance or, its equivalent, cross-correlation [86]. The correntropy represents a generalized correlation measure that includes information on both the distribution and the time structure of a stochastic process (for details see [87]).

### 3.2. Illustrations Using Model Data

Typically a dataset collected by EM has thousands of images and it is important to assess which differences are significant and to sort the images into the different populations based on these significant differences. A simple example of the classification of a set of two-dimensional (2D) images using HAC is shown in Figure 2. In this example we have a population of 12 elephants that have variable features (Figure 3(a)). For the MSA the following procedure is performed: each image of an elephant consists of *K* columns and *K* rows (Figure 3(b)). We represent each elephant from our raw dataset (Figure 3(b)) as a line of the matrix** D**, where the first row of pixels in elephant 1 represents the start of the first line in the matrix** D**, and then the density values of the second row follow the first row along the same line in the matrix. This process is repeated until all rows of elephant 1 have been laid out in the first row of the matrix (Figure 3(b)). The pixels of elephant 2 are placed in the matrix in the same way as elephant 1 but on the second line of matrix** D**. This process is repeated until all the elephants (elephant #*L*) have been added to the matrix. With just 12 pictures of elephants one can sort out the variation by three groups of features: one is related to the densities of an eye, an ear, and a tusk, the second is the front leg, and the third is the moving rear legs. How frequently these features can be observed in different images correlates with the intensity of these features in eigenvectors (or eigenimages). All eigenimages are independent of each other. The largest variations between images such as shape, size, and orientation are found in the earlier eigenimages, while those corresponding to fine details occur later on. After the calculation of eigenimages (Figure 3(c)) we can see that the first eigenvector corresponds to the average of all the elephants. In Figure 3(c) eigenimages 2, 3, and 4 reflect the variations in the presence or absence of the major variable groups of features. Appearance of these features in different contrast in the eigenimages indicates that their presence in images is not correlated since they are seen in the first four eigenimages that have nearly the same eigenvalues. Some legs are darker as they correspond to the highest variation in the position of this leg in the images of the elephants. The remaining four eigenimages have the same appearance of a grey field with small variations reflecting interpolation errors in representing fine features in the pixelated form.

At the first try of the classification (or clustering) of elephants we have produced 5 classes that were based on first four main eigenimages. Here we see four different types of elephant (classes 1, 2, 3, and 5) (Figure 3(d)). However, if we choose 10 classes, we have five distinct populations (classes 1, 2, 4, 9, and 10) (Figure 3(e)). Some classes can be repetitive; for example, 1 and 7 are nearly the same. Such small differences could be due to noise and the weight of these small vectors can have a minor role.

### 3.3. Usage of MSA for Determination of Symmetry

**Figure 4:**
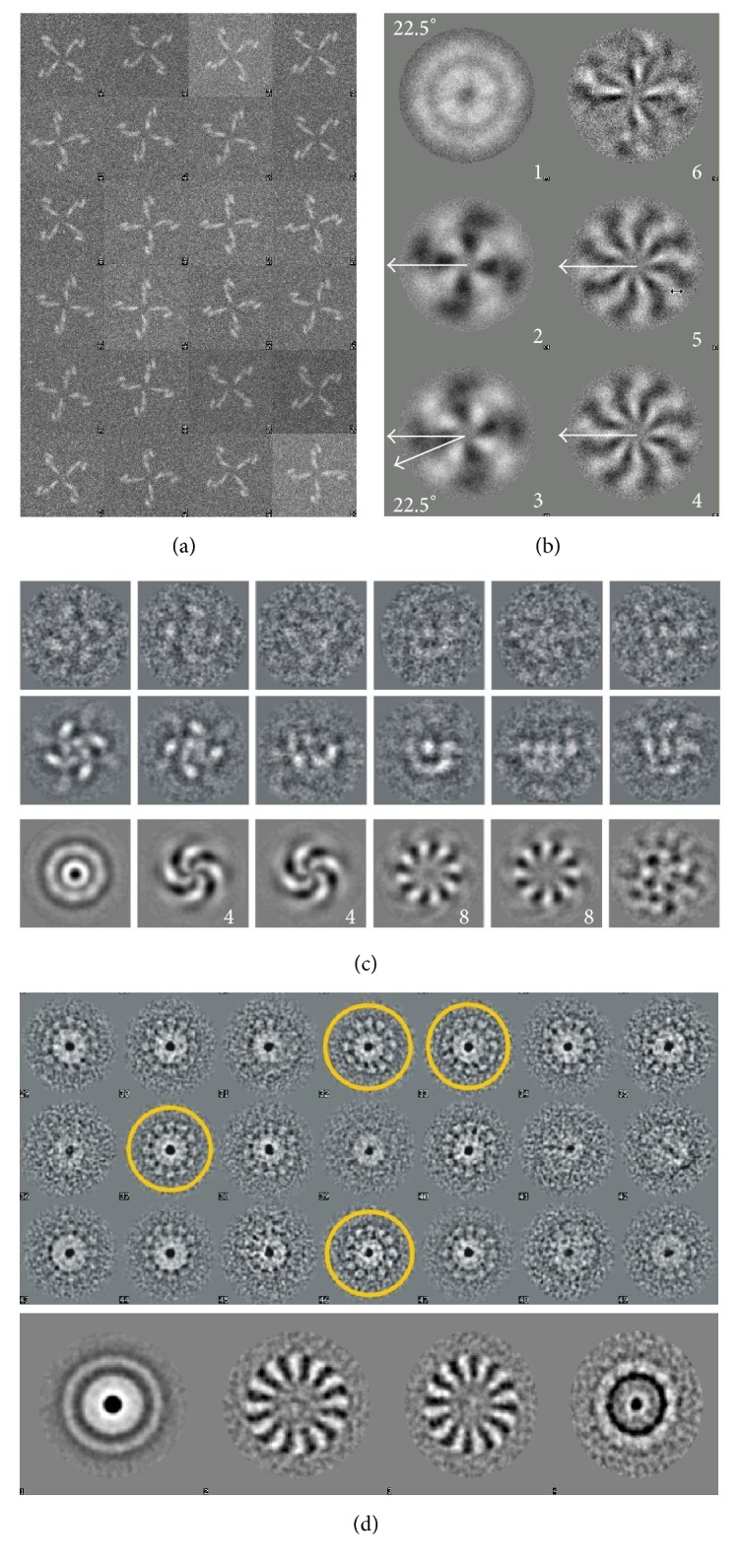
*Eigenimages-Symmetry.* (a) A model dataset with 4-fold symmetry. (b) Eigenimages from that dataset. Eigenvectors 2 and 3 have clear 4-fold symmetry and are rotated with respect to one another by 22.5° degrees. (c) Tetrameric *α*-latrotoxin raw images (top row), class averages (middle row), and eigenimages (bottom row). The eigenimages showing 4-fold and pseudo 8-fold symmetry are shown with numbers. (d) Class averages of top views from the connector of bacteriophage SPP1 are shown in the top panel. Classes where the symmetry is visible are highlighted with yellow circles. The eigenimages are in the bottom panel. Eigenimage 1 represents the total sum of the data and the 12-fold symmetry is revealed in eigenimages 2 and 3.

When doing structural analysis one has to check what sort of symmetry can be expected in the complex. MSA is commonly used to determine the rotational symmetry of complexes. Typically the rotational symmetry of a complex is only seen in its end views so these views must be separated from the side views and oblique views for this analysis to work. Even if the number of end views is not very high one can artificially increase their numbers by applying random in-plane rotations to generate more end views (Figure 3(f)). It is important to mention that the images have to be centred for symmetry analysis; otherwise the eigenimages will reflect variations due to displacements of images. In Figure 3(g) the eigenimages of the unaligned elephants shown in Figure 3(f) display some sort of featureless images with a hint of symmetry which is related to rotations of images within the frames. Eigenimages 2 and 3 look rather similar but are rotated by 90°, indicating that they are orthogonal vectors and do not provide symmetry information. However, if we look at a well centred model with 4-fold symmetry (Figure 4(a)) eigenimages demonstrate clear 4-fold symmetry (Figure 4(b)). When real data is used, for example, *α*-latrotoxin, the 4-fold symmetry is seen in the class averages and the eigenimages, calculated only for the end views (Figure 4(c), [88]). This technique also works for higher rotational symmetries as demonstrated by the connector complex from bacteriophage SPP1 (Figure 3(d), [89]). In this complex 12-fold symmetry is clearly visible in both the class averages and the eigenimages.

It is important to mention other approaches used for the determination of rotational symmetry. Crowther and Amos [90] introduced rotational power spectrum analysis of individual particles. This technique has been successfully used in many studies. However, this estimation of the symmetry is typically affected by low SNR in single images and especially for images taken in cryoconditions. Marco and coauthors described an example of the rotational symmetry assessment which uses rotational power spectra of many different end views of single particles. This is followed by a *K* nearest neighbour classification, statistical analysis with eigenvectors, and a circular harmonic analysis ([91] and references therein). These approaches are implemented in SPIDER, XMIPP, and EMAN2 [34, 41, 43].

### 3.4. Statistical Analysis of Particles of Different Sizes

**Figure 5:**
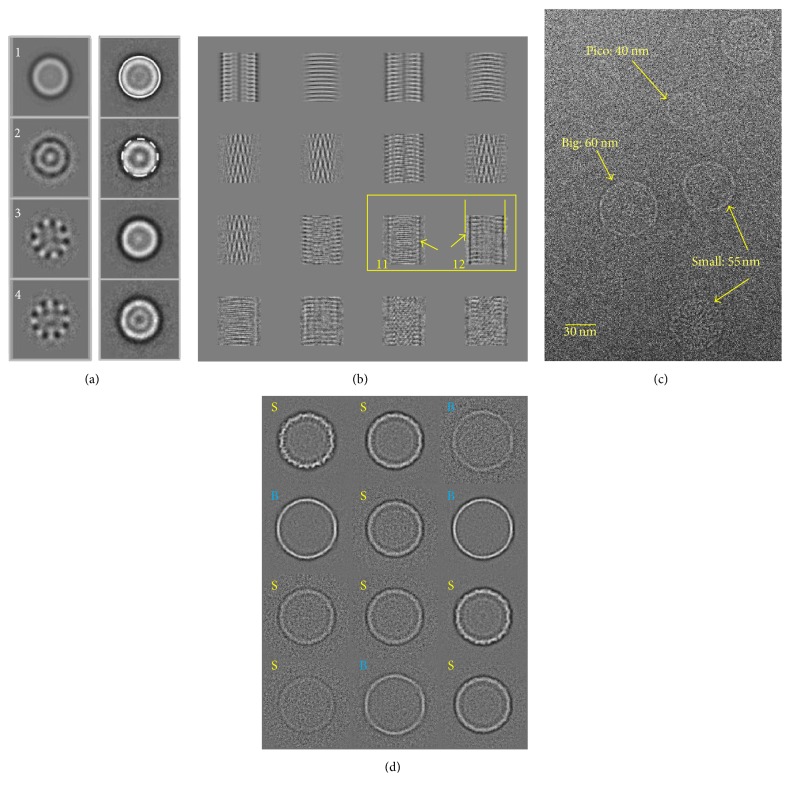
*Eigenimages-Size Variation.* (a) Eigenimages of Hsp26 are shown in the left panel. Eigenimage 1 represents the total sum of the dataset. Eigenimage 2 shows the continuous outer circle which indicates the characteristic size difference range within the dataset. The right panel shows the entire dataset separated into four classes via MSA by only using these first four eigenimages. The big class is highlighted with a white circle around its perimeter, the small class is highlighted with a dashed white circle, and the remaining two classes represent a mixture of large and small Hsp26 images. (b) Eigenimages of BSMV. The size difference is shown in images 11 and 12 (adapted from [18]). (c) A representative micrograph showing the heterogeneity of the SPP1 bacteriophage procapsids where different sizes are clearly seen [19]. (d) The classes of the procapsid images are labelled according to their size, big (B, in blue) and small (S, in yellow).

MSA is also a powerful technique for visualising size differences in a population of images. To reveal variations within a dataset related to orientation or conformational changes, the 2D images should be well aligned. The quality of the alignment can be assessed by visual examination of the eigenvectors obtained during statistical analysis. In the case of possible variations in sizes the dataset should be centred. Visual inspection of eigenvectors can indicate if the particles differ in size; in this case one can see eigenvectors with a characteristic pattern of concentric rings. The variations in overall size can be evaluated by calculation of the differences between classes and the first eigenimage (which is an average of all images). A characteristic feature of size variation in a dataset is a ring that can be seen in the second eigenimage from a dataset of Hsp26 (Figure 5(a), left panel, [33]). Then images comprising the classes with the positive difference in the outer rim (large particles) should be extracted in one subset while the images that constitute the classes with the negative outer rim (small particles) should be extracted into another subset (Figure 5(a), right panel). That will create two more homogeneous subsets. It will be natural that some differences will not show clear positive or negative outer ring that will say that the images corresponding to these classes were not separated and should be selected into the third subset and subjected to a new round of centring and subclassification. It is possible that there can be more than two size groups of molecules. This hypothesis can be verified by MSA again and images can be separated according to the eigenvectors.

Minor variations in the size of the particles are often not visible in micrographs but they limit the resolution if the particles were picked on apparent size alone and combined in the same dataset as heterogeneity would still be present. Statistical analysis has revealed that the spherically shaped molecule has two conformations, both with tetrahedral symmetry, but differing in size by about 10 Å [33]. Care has to be taken, however, that the characteristic ring is not caused by poor alignment of the molecules. The helical barley stripe mosaic virus (BSMV) also shows size variations in its eigenimages, but, rather than a circle as seen in Figure 5(a), it can be seen by the presence of black peripheral borders in eigenimage 12 (Figure 5(b), [18]).

Another example where MSA and classification has revealed variations in the size of a large complex is the study of the bacteriophage SPP1 procapsids (Figure 5(c)) where three large size differences were visible on the micrograph [19]. Alignment and calculation of eigenimages using MSA revealed minor size variations and helped to verify an improved separation (Figure 5(d)). In this case four of the classes have a size compatible with the “big” procapsid of Figure 5(c) and eight with the “small” procapsid.

MSA can also be used to assess differences in 3D structures. Sander and coworkers in 2010 used MSA to classify the aligned 3D structures of the human U4/U6.U5 tri-snRNP complex collected using Random Conical Tilt. The authors first aligned and classified all the untilted images and then calculated structures for each class using the corresponding images from the tilted micrographs. These 3D structures were aligned prior to classification. Each class contained about 5 to 8 3D structures which were calculated from 200 to 400 images. The use of MSA in this classification method allowed differences in the three main domains to be seen. Different orientations were found in the stalk of U4/U6.U5 tri-snRNP, the left head domain of the U5 subunit of tri-snRNP, and the U5 foot domain [30].

### 3.5. Statistical Analysis of Particles with Variable Ligand Occupancy

If the particles have a different composition and incomplete occupancy of a substrate, it will be useful to start from multireference alignment so that all images will be brought into orientations defined by the initial model. The images should then be separated into subsets corresponding to the more characteristic views and subjected to MSA. If a substrate has a sufficiently large mass (a component that is ≥20 kDa and not stably bound to the biocomplex) then it will be visible in the eigenvectors as localised bright or dark spots indicating local strong variations in projections. Their location in different eigenimages will depend on orientations of the particles in images. The data can be separated into subsets using the eigenvectors (images) that show the variations in question and then 3D reconstructions for each subset can be obtained, followed by assessment of the differences by calculations of difference maps [5].

**Figure 6:**
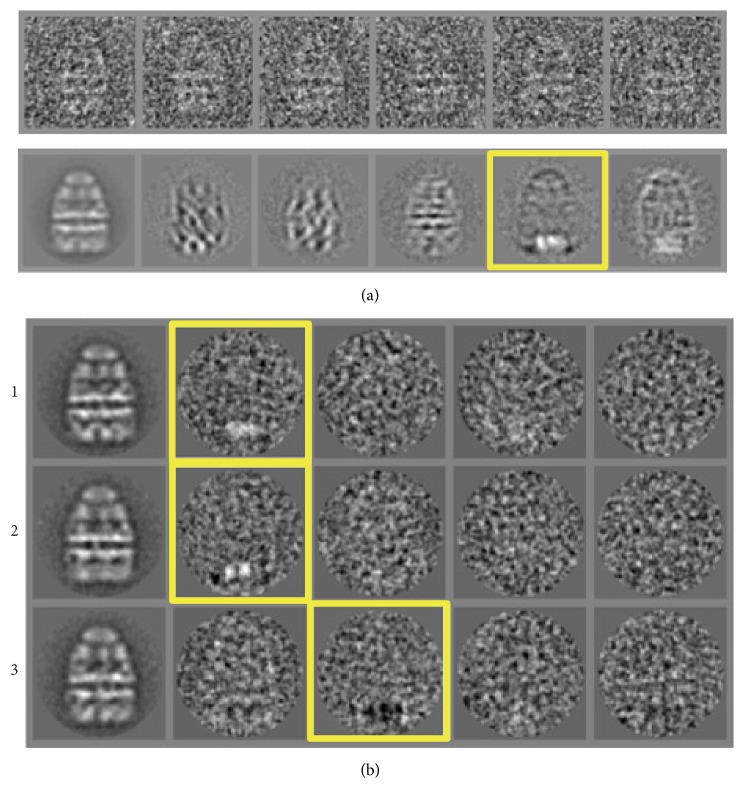
*Eigenimages-Substrate Binding.* (a) GroEL bound to the substrate rhodanese with the raw images (top) and eigenimages (bottom). Eigenimage 5, highlighted with a yellow box indicates heterogeneity in the* trans*-ring which is related to the binding of rhodanese (adapted from [5]). (b) Three of the 12 orientation classes (column 1) from GroEL-rhodanese complex after MSA based on the eigenimages, the first six of which are shown in (a). The eigenimages of these classes are shown in columns 2–5 and the heterogeneity in the* trans*-ring is highlighted with a yellow box (from [5]).

MSA was used to detect the heterogeneity in the binding of Groel-GroES-ADP with substrate rhodanese [5]. No signs of heterogeneity can be seen in the raw images (Figure 6(a), top panel), but eigenimage 5 (Figure 6(a), bottom panel) indicates, by the two bright spots in the bottom of the image, that there is variation in density in the* trans*-ring reflecting heterogeneity due to partial occupancy by the substrate. Further still, eigenimages 5 and 6 show signs of orientation variation by black and white perimeter outlines so they are not the best candidates for a separation based solely on these eigenimages. A further classification was carried out based on the first 11 eigenimages, but excluding eigenimage 5, to remove any bias towards the ligand. After this MSA, 12 classes were produced and the eigenimages obtained from these new classes showed the bright spots indicating density variation in the* trans*-ring (Figure 6(b), highlighted in yellow boxes). The data was then further classified into 3 subclasses based on the eigenimages that showed local variations in the* trans*-ring [5].

Another approach is based on the random selection of different subsets of images from the dataset and calculating a sufficiently large number of 3Ds. The statistical analysis of the 3D maps will localise the areas which have the most dominant variations of densities. Those maps showing variations in density can be used for a competitive alignment to separate the images into subsets corresponding to these 3Ds [92, 93]. Both approaches have several implementations based on slightly different algorithms and are used nowadays mainly in the structural analysis of biomacromolecular complexes.

## 4. Maximum Likelihood Estimation Method

### 4.1. Basics of ML

This approach was applied to EM studies for the first time by Sigworth [64]. The Maximum Likelihood Estimation (ML) method is used to find a model that has the highest probability of representing a dataset $I_{i}\left(\vec{r}\right)$, where *i* = 1, 2, …, *N*, and *N* is a number of images in the dataset (the approach can be applied to both 2D and 3D data). The ML method is based on the assumption that the dataset represents many copies of images of *M* structures (or images of several structures) to which noise (a general assumption that this is Gaussian noise) has been added. Our goal is to maximize the probability *P*, such that the subdataset $I_{m}\left(\vec{r}\right)$ corresponds to the model *M*_*m*_ with a set of parameters *θ*. These parameters are an estimate of the true structure, the noise, and any transformations involved.

Maximizing the likelihood is equivalent to maximizing its logarithm *L*. Assuming that individual images $I_{i}\left(\vec{r}\right)$ are independent, this function can be written as a sum of likelihood logarithms for all images $I_{i}\left(\vec{r}\right)$. This maximization is achieved by optimizing the log-likelihood function, *L*(*θ*), given by the equation [64]

(11) $$\begin{array}{c} L\left(\;\theta \;\right)=\overset{N}{\underset{i=1}{\sum }}\ln P\left(\;I_{i}\left(\;\vec{r}\;\right)\;|\;M_{m},\theta \;\right). \end{array}$$

Typically a few random images from the dataset are chosen by the user as a starting point for the analysis, sometimes referred to as “seeds.” Each particle image *I*_*i*_ in the dataset is assigned a probability that it represents a structure *M*_*m*_ and particle images with a similar probability are assigned to the same class of images *I*_*m*_.

Refinement and reassigning images to classes are based on the probability *P* that is linked to the correlation function and performed using newly assessed parameters *θ* (e.g., new angles, shifts, and correlation to projections of one of the models) with respect to the new classes obtained. An image may have good correspondence, as shown by the CCC with several projections of one model and possibly with some projections of another model. So there are several possibilities of assigning the image to one model or another. Here the probability of this image belonging to one or another model will be defined by the height of the correlation with the projections and a number of local best projections with good correspondence. The higher the CCC is an indication that the image has a higher probability *P* and that it likely corresponds to this given model. The classification is usually iterated a number of times resulting in a different quantity of particles per class each time. The number of particles chosen can be increased, so long as new information is obtained in the output class averages. It has been found that 200–300 particles per class provide a good basis for initial reconstructions, though for negative stain data fewer particles per class can be used. If there are too few particles per class, then the alignments and classification become less accurate in ML [94]. During the calculation, all particles are compared to all references in all possible orientations and weighted probabilities obtained for each case. Weighted class averages are then calculated and used as the input in the next round of optimization.

This is a slower method than a correlation based alignment but does produce good convergence. The calculation can be speeded up if prealigned particles are used and a binary mask is applied so that only areas where variations occur are included. Such masking provides an additional advantage in that the variable regions will not interfere with the area of interest and more accurate classes could be obtained. In 2007 Scheres and coworkers extended the ML method for both 2D and 3D to overcome two drawbacks: CTF had not been considered and only white noise was used [45, 46].

The ML 3D analysis requires a 3D starting model, the choice of which has a significant impact on the success of the classification. This starting model has to be determined by other methods prior to any ML classification. Often the initial model can be derived using a similar structure, either by creating a low resolution map from PDB coordinates or by using another related EM map. When this is not available, then a map can be calculated using angular reconstitution [95] or Random Conical Tilt (RCT, [96]). If RCT is used, 2D images can be classified and a 3D model calculated for each class but the missing cone of data limits the resolution obtained from this method. The 3Ds from RCT subsets can be aligned in 3D space using an ML approach where the starting reference could be Gaussian noise [97]. In order to avoid model bias, it is helpful to use a model that incorporates all the different structures in the dataset (the average one). Further complications arise if the model is not low-pass filtered. Often small details (or high frequencies) give local minima; however too many low frequencies can give blobs that will not refine. If the starting model has come from a PDB file or from a negative stain EM map, it is recommended to refine the starting model against the complete dataset; this will remove any false features and give better convergence.

A number of models or “seeds” are needed for the ML 3D classification as it is a multireference alignment. If four starting seeds are used, then the whole dataset can be divided initially into four random subsets and each one refined against the starting model created from the PDB, EM, or other method. As in 2D classification, the number of seeds has to be chosen carefully and should correspond approximately to the expected possible conformations of structures, but their number may be limited by the size of the dataset or computing power available. Hierarchical classification can also be used. For example, an initial classification into four classes of a ribosome dataset gave two intact and two broken structures. The particles in the intact classes were then separated into four more classes, which showed two classes with strong RNA density while the other two did not have any tRNA densities corresponding to tRNA. The two classes with strong tRNA density were further classified into four more classes, and these four classes showed alternative tRNA conformations [94].

ML is a computationally expensive procedure and Scheres and coauthors [65] introduced a faster search algorithm by reducing the search space. Since the assignments of *K*_*l*_ and *θ* are independent, the probability of assigning image *I*_*i*_ to the reference *K*_*l*_ can be evaluated by summation of probabilities over a range of possible rotations and translations of *I*_*i*_ during the first iteration of the examination; all translations are saved in the data file of processing results. Reduction of the calculation time can be achieved by further iterations, if the probability of *I*_*i*_ to be assigned to *J*_*l*_ is not significant, and then it is assumed that none of these translations will increase the probability that the image corresponds to the reference images used in the next iteration. Therefore integration over the translations is not performed. Scheres with coauthors [65] obtained almost identical results using this fast method compared to the full search, but the fast method was 6.5 times faster when compared to the full-search protocol. Nonetheless, in all cases where ML is used, care must be taken in choosing the search space to avoid being trapped in a local minimum. An overview of maximum likelihood has been given by Scheres [94] and Sigworth with coauthors [98].

### 4.2. Examples of Usage of ML in Analysis of Heterogeneity

This technique has been used for a variety of different complexes in EM. Lee et al., 2011 [99], applied the technique to helical objects: firstly to a homogeneous dataset of TMV which had one class and secondly to a NaK ion channel. The NaK ion channel had two classes, each with a different helical symmetry, and resolutions of 7.84 Å and 7.90 Å were obtained. Wang and coauthors [71] were able to resolve conformational changes in viruses using time resolved experiments. The structures have shown different stages of the maturation of Nudaurelia Capensis Omega Virus, an RNA virus. This virus had been previously studied using difference maps [100] but this procedure restricted the difference to a small region of the structure. However, the use of maximum likelihood allowed the authors to view more steps during the maturation process.

**Figure 7:**
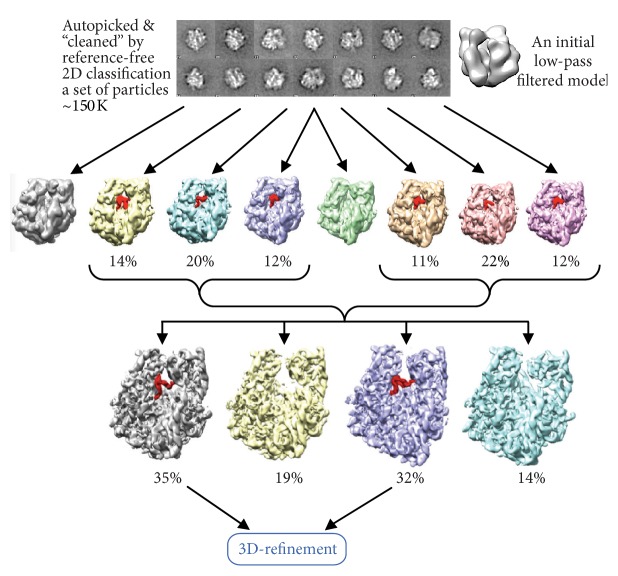
ML procedure in the analysis of conformational changes of biocomplexes. Raw images are firstly assigned initial orientation angles using the initial model. That is typically done by projection matching. Then the ML approach is used to obtain 6 to 8 reconstructions. Each 3D model is visually examined in the area of interest; for a ligand presence, in this case the bound tRNA is highlighted in red. Images which were used to obtain the models with tRNA are extracted and subjected to the next round of classification. The following step involves extracting images corresponding to one or another conformation and then followed by refinement. The percentages below the structures in the top row indicate fractions of images from the entire dataset used to calculate these models, while in the second row the percentages are taken from the number of images supposedly containing the bound tRNA.

RELION implements a modified version of ML, where the adaptive expectation maximization algorithm is used thus allowing faster processing [37]. The algorithm has been described by Tagare et al. [101]. RELION is successfully used in the analysis of conformational changes of large biocomplexes. This approach is based on a few major steps. Firstly data cleaning is performed by 2D classification for the removal of bad particles which do not correspond to the fully assembled complexes or badly misaligned images. Images which belong to bad classes are eliminated from further processing. Then the 3D ML classification is applied to the cleaned dataset and typically 2 to 8 structures are produced. These maps are then examined in the designated areas for the presence of any expected ligands and for the case of the ribosome this would be elongation factors or different tRNAs (Figure 7). Images which were used to obtain structures with similar features are extracted into separate subsets and subjected to the next round of 3D classification. Subseparation of the dataset allows one to distinguish different states of large biocomplexes and refine their structures to high resolution [15, 102, 103].

ML has also shown to be effective in tomography. Scheres and coauthors [47] first tested their approach on GroEL and GroEl-GroES models. Electron density maps were calculated at 2.5 nm resolution from PDB coordinates of GroEL and GroEl-GroES. Images of GroEL and GroEl-GroES were randomly selected from all datasets and 200 subtomograms were calculated. Three classes were obtained using a maximum likelihood approach combined with unsupervised alignment followed by classification. Two classes showed 7-fold symmetry, one class contained GroEL, and one contained a GroEL-GroES complex, while the third class could not be assigned to either GroEL or GroEL-GroES. Scheres and coauthors [47] then extended their method to a p53 mutant in complex with dsDNA starting with only 40 RCT reconstructions. The two averaged models obtained the following: the structure with C2 symmetry was similar to an independent reconstruction using common lines. A structure without any imposed symmetry differed from the C2 structure by a movement in the top part of the structure.

## 5. *K*-Means Clustering

**Figure 8:**
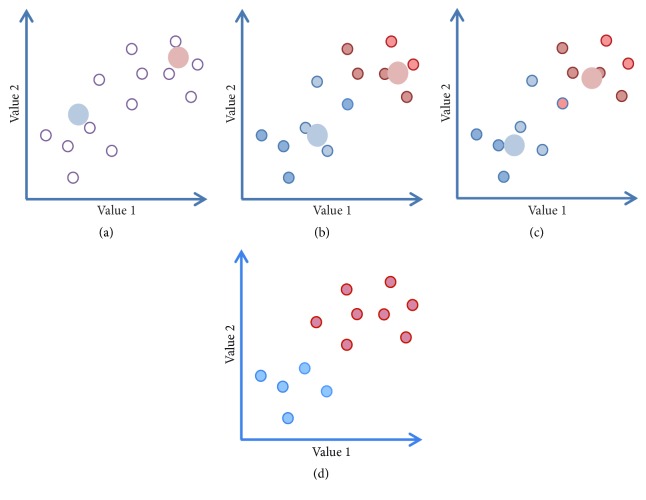
*K-Means Clustering.* (a) Two initial seeds are randomly placed within the data. (b) Step  2 indicates positions of the averages of images that are nearest to the seeds. (c) The averages are then recalculated based on the assignments in step  2. Steps  2 and 3 are reiterated; (d) shows the final classes.

*K*-means clustering is used to separate the image data into a number of possible structural conformers. Centroid-based* K*-means clustering is based on the concept that there is a central vector, which may not necessarily be a member of the dataset, around which the subdata can be grouped. The number of clusters is user defined, for example, to *K*; the initial *K* seeds are set typically randomly (Figure 8). The optimization task is to find such *K* centres of clusters, such that the data objects (images) of a class (cluster) will be located to the nearest cluster centre [63]. If we have a number of images (*I*_1_, *I*_2_, …, *I*_*N*_), where each image is a* d*-dimensional real vector (see above in the MSA section),* K*-means clustering aims to separate the *N* images into *K* subsets, where *K* ≪ *N* and *I*_*n*_ ∈ {*S*_1_, *S*_2_,…, *S*_*K*_}. Separation of images *I*_*i*_ into subsets *S*_*k*_ is based on the minimization of within-cluster sum of squares (WCSS) (sum of distance functions of each point in the cluster to *K*_*k*_ centre). Therefore a set of observations (our data *I*_*i*_) is divided into a series of subsets *S*_*k*_, under the constraint that the variance of the WCSS should be minimized. In other words, its objective is to find the minimum arg⁡min_*s*_⁡  of possible distances between a centre and data elements (images):

(12) $$\begin{array}{c} \underset{s}{\arg\min}\;=\overset{K}{\underset{k=1}{\sum }}\underset{I\in S_{k}}{\sum }{\left\|\;I_{k\;avr}-I_{i}\;\right\|}^{2}, \end{array}$$

where *I*_*k* avr_ is the mean of images in the class *S*_*k*_. The proximity between images *I*_*k* avr_ and *I*_*i*_ is estimated by the distance between the end points of the vectors (Euclidean distance).

The first step assigns each image to the cluster that gives the smallest WCSS with respect to the chosen seeds. So nearest neighbours are first ranked and counted, and then a class membership assignment is made and an initial class averages are defined. This is illustrated in Figure 8(a) where a set of particles are randomly put into 2 clusters. The average of each cluster is calculated (Figure 8(b)) and the centroids of these new clusters are taken to be the new mean and the assessment of the distances is repeated. The particles are reassigned according to which centre is the nearest to them, shown as a solid circles in Figure 8(c). This two-step process continues until there is no change in where the observations are assigned and convergence is therefore achieved (Figure 8(d)). The Euclidean distance is commonly used to assess a level of similarity (closeness) between images, but it is typically affected by noise in images. Normalization and dimensionality reduction like the coarsening of data are helping to improve the quality of clustering and speed up the calculations.

More recently new approaches where the distance metric learning from training data is used improve the prediction performance of *K*-means clustering methods [70]. Recently Extended Nearest Neighbour (ENN) Method for pattern recognition has been described where the distance-weighted approach is used. Improvement of the efficiency in ENN is achieved by a preprocessing step where a subset (randomly selected) of the dataset is used to make a classification decision. Then all elements in the dataset are ranked according to the distances from the initial classes and assignment to a class is done to maximize the intraclass coherence [104].

## 6. Three-Dimensional Covariance

MSA and ML methods are widely used for both the global quality assessments of images (or maps) and for the examination of local variations. Such information on local, real-space, differences between the maps is essential for understanding if the changes are related to different conformations or due to noise. Assessment of the 3D variance between multiple 3D structures provides an effective tool to assess the stability of each element in the structures. In the covariance matrix used in EM, a single row contains the covariance between voxels of one volume with the corresponding voxels of another volume. If the voxel is located in the area of a ligand that is present in all maps, the matrix will show large covariance of this ligand area with the ligand areas in other maps, but if in other structures ligand is absent then the covariance will be weak and that will indicate that there are changes caused by unstable ligand binding. However, the local differences revealed by the value of voxel-by-voxel real-space variance may arise from errors in the reconstruction procedure such as bad alignments or an uneven distribution of angles defined for the images [68].

Different methods have been proposed to estimate the covariance matrix. Penczek and coauthors [28] used bootstrapping to calculate the covariance of many volumes. By its nature bootstrapping assumes that subsets of images are randomly selected from the dataset and that 3D is generated from each subset [28, 92]. Sometimes, bootstrapping can produce wrong correlations in the resampled volumes due to multiple duplicates between subsets. This occurs if the Euler angles are not evenly distributed and the structural features became distorted. If there are only small differences between structures or, in the case where there are no discrete conformations, current reference-free classification schemes may not always be effective. In order to overcome these problems, techniques that examine the information inside the covariance matrix are being developed. A major obstacle in this approach is the large size of the matrix that should be analysed for major variations. To make the process of calculation faster it was suggested that the 3D maps should be coarsened [65].

The calculations of 3D variance of maps help to find the arias with high variations. The covariance of a 3D map indicates how variations in the density at one voxel correlate with variations in another voxel. Conformational changes where a structural element is found in different positions in two structures would come from a negative covariance between these two locations in the map. Calculations of the covariance of maps is computationally highly demanding (the covariance matrix of a 10^6^-voxel map will have 10^12^ entries) but techniques have been developed recently to identify the principal components of the covariance [28, 49, 105]. Anden and his collaborators [39] optimized the algorithm by using a conjugant gradient method. The conjugate gradient method is an iterative algorithm, allowing the best approximation of the solution of large systems of linear equations to be found [106]. This has the advantage of allowing a nonuniform distribution of angles where the CTF can be taken into account.

## 7. Bootstrapping

**Figure 9:**
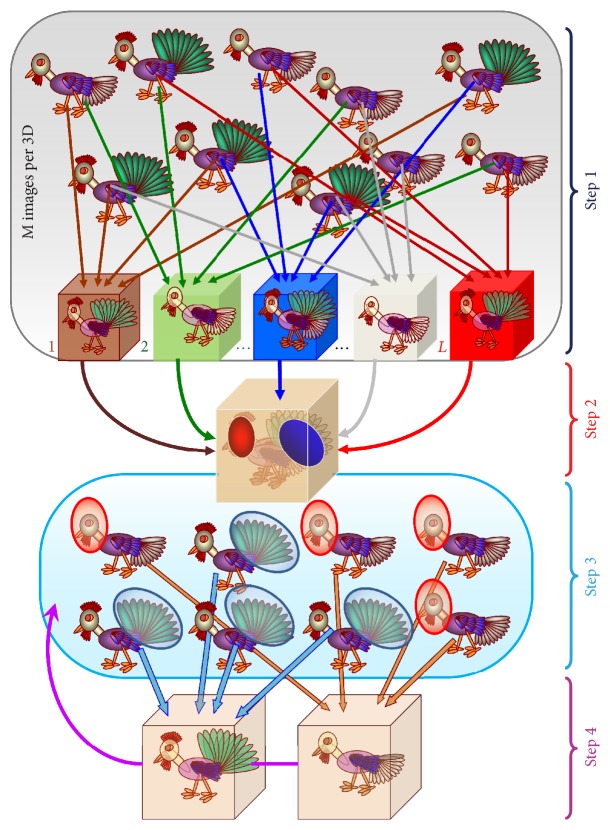
*Bootstrapping.* A representative set of chickens with different tails and head positions. During step one each of *L* subsets of M images was picked to make *L* reconstructions. During step  2 the variance within *L* reconstructions determines the most significant differences in the head (green) and tail (red) positions. The result of the classification of images shown in step  3 is done by analysing the level of variance in areas defined in step  2 (highlighted by red and blue circles). The two reconstructions generated are then used as the input to carry out the refinement using the focused classification (step  4).

In bootstrapping a number of data subsets, referred to as a “resample,” are selected from the original large dataset, where each subset contains the same number of images although images can be duplicated both within one subset and between subsets (Figure 9). In the next step reconstructions from each subset are calculated and the voxel-by-voxel variance of these maps is calculated yielding an estimate of the overall variance distribution. That allows assessing the differences between the cryo-EM maps: the magnitude of the variance in cryo-EM maps is used to identify areas of high variance. This information can then be used to sort a heterogeneous dataset and obtain 3D structures for the different conformations [92].

This procedure can be illustrated with an example of chickens that have different head positions and different tails. A subset of data consists of the images taken from the original set by selecting some images. Several subdatasets (Figure 9) contain the same number of chickens but differ in the number of each conformation within the subsets. During the first step of the bootstrap procedure the entire dataset of EM images that represents a set of 2D projections of several structures is separated into many subsets and for each a 3D map is calculated (Figure 9). All the 3D maps are low-pass filtered and the variance and covariance of the mean between them are calculated and a cross-correlation coefficient is obtained. The resampling process is then repeated many times and a mean calculated every time, each one being called the bootstrap estimate. A histogram of these means will indicate how much the mean varies. Areas of variance in the maps can then be visualised. In Figure 9 (step  2) the red and green spheres correspond to the variations in head position and tail type, which are highlighted in the model below. The following step of the procedure involves masking out areas surrounding the region of interest with high variance. These masks are then projected at different angles, determined for the images, producing a set of 2D masks which are then used to eliminate stable features and classify the 2D images according to variations of the selected region. In step  3 all the 2D images are sorted into subsets according to their Euler angles and a* K*-means clustering is used for each subset in the areas of variance determined from the 2D masks. The number of groups obtained for each set usually corresponds to the number of different structures expected. A multireference competitive 2D alignment is performed against 2D projections of the models obtained in step  4 allowing for structure refinement. Then the corresponding new 3D structures are calculated. The refinement is performed iteratively with images corresponding to these new 3D structures until one ends up with structures for each conformation (Figure 9).

This method was used for single-particle analysis of* E. coli* 70S ribosome complex with tRNA and elongation factor G (EF-G) [36], after a variance map indicated some highly variable regions. A bootstrapping method was used to elucidate two different structures: one with bound EF-G and an empty A site, while the other had no EF-G but had tRNA in the A and O sites. Zhang et al., 2008, implemented a bootstrap technique using Fourier methods that also corrected for the Contrast Transfer Function (CTF). The authors created two sets of 2D projections from a test object: one set from a structure that was CTF corrected and another without CTF correction. The next step included the standard bootstrap procedure of calculating a number of different maps for each dataset followed by calculations of two 3D variance maps. The maps from the CTF corrected 3D produced the variance found originally, but the map from the uncorrected data had strong artefacts making it difficult to find regions of real variance. This computational experiment indicates the importance of the CTF correction for the improvement of a resolution in structures [68].

Liao and Frank [35] proposed an approach for separation of different conformations using the bootstrap technique and tested it on an* E. coli* 70S ribosome dataset (previously been subjected to the ML technique, [45, 46]). They used five eigenvolumes and looked for two classes. The difference in the two structures was immediately obvious as EF-G was visible in one map but not on the other and the L1 stalk was in a different position in both maps. Penczek with coauthors [28] analysed the stalk area of a 70S *∗*tRNA*∗*EF-Tu*∗*GDP*∗*kirromycin ribosome complex and found four separate structures: two represent the main conformation with or without the E-site tRNA while the others show the rotated conformation with a P/E hybrid site tRNA. Simonetti et al., 2008 [93], determined the structure of a 30S ribosome initiation complex with tRNA and fMet-tRNA^fMet^ and initiation factors IF1 and GTP-bound IF2. They found five different structures that were statistically relevant ranging from 8% to 40% of the dataset. All structures contained tRNA and fMet-tRNA^fMet^ and IF1; however, the conformation of fMet-tRNA^fMet^ was different in the structures where 1F2 was absent.

## 8. Neural Networks

An artificial neural network (NN) is a concept, based upon the NNs in animals, particularly in the brain, and is used to estimate functions with a large number of inputs and classify them into certain groups. A self-organizing map (SOM) algorithm [107] appeared to be efficient in image analysis. The dataset of EM images represent the input for the self-organizing map (network). Here it is assumed that the dataset of images are represented as vectors $I_{i}\left(\vec{r}\right):I_{i}\in R^{n}$, where *i* is an index of the image within the dataset sequence and there is a set of variable reference vectors (in our case a set of images) $M_{m}\left(\vec{r}\right):M_{m}\in R^{n}$, where *m* = 1,2,…, *J*. *J* is the number of references. At the starting point the references $M_{m}^{0}\left(\vec{r}\right)$ can be selected randomly as some images form the dataset. Sequentially each image $I_{i}\left(\vec{r}\right)$ is compared with each reference $M_{m}\left(\vec{r}\right)$. The comparison could be based on the assessment of the Euclidean distance between the image and the reference:

(13) $$\begin{array}{c} d\left(\;I,M\;\right)=\left\|\;I_{i}\left(\;\vec{r}\;\right)-M_{m}^{0}\left(\;\vec{r}\;\right)\;\right\| \end{array}$$

and the best reference $M_{m}^{0}\left(\vec{r}\right)$ corresponding to this image *I*_*i*_ with min⁡ (*d*(*I*_*i*_, *M*_*m*_^0^))⁡ will be modified for the analysis of the next image:

(14) $$\begin{array}{c} M_{m}^{t+1}\left(\;\vec{r}\;\right)=M_{m}^{t}\left(\;\vec{r}\;\right)+\alpha_{m}^{t}\left[\;I_{i}\left(\;\vec{r}\;\right)-M_{m}^{t}\left(\;\vec{r}\;\right)\;\right], \end{array}$$

where 0 < *α*_*m*_^*t*^ < 1 is a coefficient that defines the amplitude of the correction and is linked to the references and decreases during following iterations, and *t* is a number of an iteration. The output nodes are elements of a 2D array with an image associated with each node. The node $N_{m}^{t}\left(\vec{r}\right)$ of the data is obtained by summation of all images $I_{i}\left(\vec{r}\right)$ that are closest to the reference $M_{m}^{t}\left(\vec{r}\right)$ during iteration *t*. That is done using the weighting function *W*_*j*_^*t*+1^(*R*) where *R* is the distance between nodes:

(15) $$\begin{array}{c} N_{m}^{t+1}\left(\;\vec{r}\;\right)=M_{m}^{t}\left(\;\vec{r}\;\right)+W_{m}^{t+1}\left(\;R\;\right)\alpha_{m}^{t}\left[\;I_{i}\left(\;\vec{r}\;\right)-M_{m}^{t}\left(\;\vec{r}\;\right)\;\right]. \end{array}$$

This node is then used to create a centre in a neighbourhood of nodes within a defined radius. A comparison of the entire dataset is repeated during the iteration *t* + 1 with modified references and the nodes will also be updated until the process converged. This is a simplified explanation of basic principles of SOM.

Marabini and Carazo [108] introduced the concept of SOM to NN in EM. Marabini and Carazo [108] found the method to work not only on rotationally misaligned homogeneous data revealing different orientations of biomolecules but also on aligned heterogeneous data. Pascual-Montano et al., 2001 [48], introduced a further self-organizing map which they called KerDenSOM (kernel probability density estimation self-organizing map). Here they describe each step in a more laborious way than that proposed by Kohonen [107]. This method has been used in sorting areas extracted from 3D tomographic maps [109]. A mask was applied to extract cross-bridge motifs in 3D tomographic maps from Insect flight muscle in a rigor state, which were then subjected to a multireference alignment prior to being subjected to SOM. KerDenSOM needs aligned motifs to successfully extract the structural differences in the dataset. A large rectangular output map provides a better separation of classes than a square map as data in high dimensions tends to have an ellipsoidal rather than a spherical shape [48].

Classification can be done using rotational power spectra of the images rather than the images themselves. This has often been used in conjunction with neural networks using the KerDenSOM map. Pascual-Montano et al., 2001 [48], tested their algorithm on rotational power spectra of negative stain images from the G*40*P helicase of* B. subtilis* bacteriophage SPP1. Núñez-Ramírez et al., 2006 [110], used the rotational power spectra of images from the replicative helicase G40P to determine the structures of three different hexamers. The initial power spectra were classified using the KerDenSOM algorithm [48] and could then be sorted into three datasets according to the symmetry: 3-fold, 6-fold, and 6-fold with a 3-fold contribution. After sorting data into different subsets, it is always advisable to check how homogeneous the datasets are. Núñez-Ramírez et al., 2006 [110], checked the homogeneity of their three subsets using KerDenSOM on both the images and the rotational power spectra of the images.

## 9. Conclusions

There are many techniques that can be used in the analysis of heterogeneous data; however, each biological dataset will often require a very specific method to resolve the problem. The different statistical methods, examples of which have already been described, are often used in conjunction with each other (Table 1). Peña with collaborators [111] used the ML method and a NN self-organizing map to align and classify the dataset of the full-length hexameric TrwK, a VirB4 homologue, in the conjugative plasmid R388. This molecule consists of two rings, one with a diameter of 132 Å corresponding to the N-terminal region of the protein and one with a diameter of 124 Å from the C-terminal region.

Pascual-Montano et al., 2001 [48], also used ML classification and NN (SOM) to look at the variability of negative stain images of the G40P helicase of* Bacillus subtilis* bacteriophage SPP1 and cryoelectron images of the Simian Polyomavirus SV40 large T-antigen. This combination of approaches (ML and SOM) used power spectra to determine symmetry of G40P particle images and has demonstrated the presence of three types of particles, one with 4-fold symmetry and another with 5-fold symmetry and asymmetric particles possibly which were not very well aligned. Analysis of results has suggested that images belonging to the asymmetric group should be removed completely from the data for further analysis. Using the techniques mentioned above the images of the SV40 large T-antigen revealed the existence of several classes of particles. Some of these particles exhibit axial curvature along the major vertical axis.

When E1 helicase was labelled with FAB antibodies, only about 30% of the particles had antibody bound to it. It was difficult to see any differences in images since the intensities related to the FAB were minimal. To overcome this problem a 3D bootstrapping technique was employed in combination with 3D MSA [112]. The first 10 eigenvectors demonstrate the variations of density distribution in the area of the FAB position.

The existence of many different software packages provides a variety of options to electron microscopists for analysis of their data (Table 1, [102, 113–117]). The packages only partly overlap and it would be useful to combine the best features of each software program. But these packages have different data formats; for example, the output file from IMAGIC needs to be converted into SPIDER format prior to the use of that program. Therefore one should take care of the data consistency between the packages. Nowadays there are several packages (EMAN2, IMAGIC, BSOFT, and some others) that can do that easily.

EM is presently obtaining structures at high resolution on a regular basis. Increasing computational power and multiprocessor technology allows millions of images to be processed. Biocomplexes in solution naturally have different conformational states and these are all captured at the same time during cryo-EM imaging. The presence of heterogeneity means that higher resolution features could be averaged out during the reconstruction phase. To avoid this problem we need to take care of “computational purification” of the entire dataset and hence the separation of data is required into more homogeneous subsets. Researchers are constantly developing new computational techniques for sorting heterogeneous datasets and extending the current approaches to more complex problems. Accurate structure determination is important in understanding the structure/function relationship of biological processes. Biological processes are not static but the components are in constant natural motion. Therefore, to understand the interactions, their sequence, and how they can be controlled, especially in the case of diseases, we need to capture different conformational states of biocomplexes. Consequently, much data collected now is heterogeneous and the methods described here as well as their applications are becoming increasingly significant. Table 1 lists some of the packages available, the methods implemented in them, and some examples of their usage. The reader has to take into consideration that it is difficult to provide a complete overview of all methods presently developed, but we hope to provide the readers with a starting point for their analysis and the ability to extend the approaches they use to obtain accurate final structures.

## Figures and Tables

**Table 1:** Packages used to work with heterogeneous datasets.

Package name	Package reference	Examples	Statistical Method used	References
IMAGIC	Van Heel et al., 1996 [32], https://www.imagescience.de/imagic_em.html	Hsp26 GroEL-Rhodanese 70Sribosome and the U4/U6.U5 tri-snRNP	MSA	White et al., 2004 [33] Elad et al., 2008 [5] Sander et al., 2010 [30]

SPIDER	Frank et al., 1996 [34], http://www.wadsworth.org/spider_doc/spider	70S ribosome 70S ribosome, tRNAs & elongation factor G (EF-G) complexes	MSA BS	Liao and Frank, 2010 [35] (for bootstrapping volume calculation) Penczek et al., 2006 [36]

RELION	Scheres, 2012 [37] Scheres, 2015 [38], https://www2.mrc-lmb.cam.ac.uk/relion/index.php/Main_Page	70S ribosome complex Ltn1 E3 ligase (four model refinements) Subtomogram averaging	ML (regularized likelihood optimization), BS	Anden et al., 2015 [39] Lyumkis et al., 2013 [13] Bharat and Scheres, 2016 [40]

EMAN2	http://blake.bcm.edu/emanwiki/EMAN2	*Beta-galactosidase* GroEL, *Ca2+ release channel*	MSA, BS, CM	Tang et al., 2007 [41], https://www.youtube.com/c/SteveLudtke

SPARX	Hohn et al., 2007 [42], http://blake.bcm.edu/emanwiki/EMAN2	70S ribosome 70S ribosome Ltn1 E3 ligase (single model refinement)	BS,CM	Liao and Frank, 2010 [35] (for eigendecomposition) Penczek et al., 2011 [28] Lyumkis et al., 2013 [13]

XMIPP	Sorzano et al., 2004 [43] De la Rosa-Trevín et al., 2013 [44], http://xmipp.cnb.csic.es/	70S ribosome & Simian Virus 40 large T-antigen groEL/groES complexes & p53 *B. Subtilis* G40P helicase & SV40 large T-antigen	ML NN	Scheres et al., 2007 [45, 46] Scheres et al., 2009 [47] Pascual-Montano et al., 2001 [48]

ASPIRE	(http://spr.math.princeton.edu/)	70S ribosome	CM	Katsevich et al., 2015 [49]

FREALIGN	Grigorieff 2007 [50] http://grigoriefflab.janelia.org/frealign	70S ribosome	ML	Lyumkis et al., 2013 [51]

Appion	http://www.appion.org	integration of different software packages		Lander et al., 2009 [52]

Scipion	http://scipion.cnb.csic.es	shell that combined different packages		de la Rosa-Trevín et al., 2016 [53]

MSA: multivariate statistical analysis; ML: maximum likelihood; BS: bootstrapping; CM: covariance maps; NN: neural networks. For more software packages see http://www.emdatabank.org/emsoftware.html.
